# Multicenter Study of Colistin Heteroresistance in Carbapenem-Resistant Klebsiella pneumoniae Strains in China

**DOI:** 10.1128/spectrum.02218-22

**Published:** 2023-07-10

**Authors:** Yuesong Weng, Tao Wang, Bin Huang, Hua Yu, Wei Jia, Bin Shan, Fen Qu, Yiwei Tang, Liang Chen, Hong Du

**Affiliations:** a Department of Clinical Laboratory, The Second Affiliated Hospital of Soochow University, Suzhou, Jiangsu, China; b Department of Clinical Laboratory, The Affiliated Peoples' Hospital of Ningbo University, Ningbo, Zhejiang, China; c Department of Laboratory Medicine, Ningbo First Hospital, Ningbo, Zhejiang, China; d Center of Clinical Laboratory, The First Affiliated Hospital of Soochow University, Suzhou, Jiangsu, China; e Department of Laboratory Medicine, The First Affiliated Hospital of Sun Yat-sen University, Guangzhou, Guangdong, China; f Sichuan Academy of Medical Science and Sichuan Provincial People’s Hospital, Chengdu, Sichuan, China; g Center of Medical Laboratory, General Hospital of Ningxia Medical University, Yinchuan, China; h Department of Laboratory Medicine, The First Affiliated Hospital of Kunming Medical University, Kunming, Yunnan, China; i Laboratory Medicine Center, Aviation General Hospital, Beijing, China; j Department of Medical Affairs, Danaher Diagnostic Platform/Cepheid (China), New York, New York, USA; k Hackensack Meridian Health Center for Discovery and Innovation, Nutley, New Jersey, USA; l Department of Medical Sciences, Hackensack Meridian School of Medicine, Nutley, New Jersey, USA; Memorial Sloan Kettering Cancer Center

**Keywords:** CRKP, colistin, heteroresistance, antibiotic resistance

## Abstract

Colistin has been considered a last-line option for the treatment of infections caused by carbapenem-resistant Klebsiella pneumoniae (CRKP). Heterogeneous resistance to colistin leads to unexplained clinical colistin treatment failure for CRKP. Our study aimed to investigate the extent of colistin heteroresistance among CRKP strains in China. A total of 455 colistin-susceptible strains, collected from six tertiary care hospitals in China, were characterized. The overall rate of colistin heteroresistance was 6.2%, as determined by the population analysis profiles (PAPs). Genomic analysis revealed that 60.7% of the colistin-heteroresistant isolates belonged to the epidemic sequence type 11 (ST11) clone. Single-nucleotide polymorphisms (SNPs) suggested that 6 ST5216 strains shared the same origin. Each of the subpopulations had a ≥8-fold decrease in colistin MIC in the presence of carbonyl cyanide *m*-chlorophenylhydrazone (CCCP), which indicated that heteroresistance could be suppressed by an efflux pump inhibitor. In addition, our results suggested that the PhoPQ pathway plays an important role in the mechanisms of heteroresistance.

**IMPORTANCE** The problem of CRKP has raised alarms concerning global health. Our study enriches the epidemiological study of colistin heteroresistance among CRKP strains in China, where the prevalence of this phenomenon was previously unknown. Importantly, colistin-heteroresistant strains may cause the failure of clinical treatment with colistin, even if the clinical laboratory reports that the strains are sensitive. The commonly used broth microdilution method is unable to detect this special phenomenon. Additionally, our results indicate that efflux pumps play a major role in colistin heteroresistance, and inhibitors can effectively reverse it. Our study is the first to provide a detailed analysis of the prevalence of colistin heteroresistance in China, as well as an analysis of the genetic mechanisms of this phenomenon.

## OBSERVATION

Klebsiella pneumoniae strains frequently cause community-acquired and nosocomial infections such as pneumonia, urinary tract infections, liver abscesses, and bloodstream infections (1). Increased antimicrobial resistance in K. pneumoniae can be the result of improper or excessive use of antibiotics in clinical settings and in the community. Carbapenems are considered one of the last lines of defense against serious multidrug-resistant K. pneumoniae infections; in recent years, however, carbapenem-resistant K. pneumoniae (CRKP) strains have been increasingly detected worldwide (2–4). Treatments for infections caused by CRKP are limited, while tigecycline and colistin are used as some of the few currently available therapeutic drugs (5). Colistin remains a highly efficient antimicrobial with activity against CRKP. However, colistin resistance, including heteroresistance, has recently emerged worldwide.

Heteroresistance is an antibiotic resistance phenomenon in which bacterial strains are composed of a minor resistant subpopulation and a major susceptible subpopulation, with the resistant subpopulation being able to replicate rapidly in the presence of the antibiotic (6, 7). This resistance mechanism was first reported for K. pneumoniae in 2008, and it has subsequently been described for other Gram-negative bacteria (8, 9). However, routine clinical colistin susceptibility testing methods, such as broth microdilution (BMD) and the Etest, were unable to detect colistin heteroresistance (10). Currently, the status of colistin heteroresistance in clinical CRKP strains remains largely undetermined. Here, we performed a large-scale multicenter retrospective study to investigate colistin heteroresistance in CRKP strains in China.

A total of 455 unduplicated CRKP strains were collected between 2016 and 2019, in a multicenter carbapenem-resistant *Enterobacterales* surveillance study in China, from six tertiary care hospitals, including hospitals in Suzhou (eastern China), Beijing (northern China), Guangzhou (southern China), Chengdu (southwest China), Kunming (southwest China), and Ningxia (northwest China). CRKP was defined on the basis of imipenem or meropenem MICs of ≥4 mg/L, as determined with a Phoenix M50 automatic microbiology analyzer. The colistin MICs were determined by the BMD method according to Clinical and Laboratory Standards Institute recommendations (11) and interpreted using the European Committee on Antimicrobial Susceptibility Testing (EUCAST) breakpoints (https://www.eucast.org/clinical_breakpoints) for *Enterobacteriaceae* (susceptible, ≤2 mg/L; resistant, >2 mg/L). The colistin susceptibility testing was performed in duplicate on two different days. Escherichia coli ATCC 25922 was used as the quality control (QC) strain. Susceptibility testing confirmed that 455 CRKP strains were susceptible to colistin.

To detect colistin heteroresistance, the population analysis profile (PAP) method was used, as described previously (6). Briefly, each isolate was grown from a single colony overnight in lysogeny broth (LB) containing 0.5 mg/L meropenem, and serial 10-fold dilutions were plated on solid LB agar with colistin at the concentrations of 0, 0.5, 1, 2, 4, 16, 32, and 100 mg/L. The frequency of colistin heteroresistance was calculated as the number of bacterial colonies that grew on the LB plates containing colistin divided by the number of bacteria that grew on the LB plates. Colistin heteroresistance was defined if the frequency with 16 mg/L colistin was at least 1 in 10^6^ colonies but less than 5 in 10^1^ colonies (6). Heteroresistant subpopulations grown with the highest concentration of colistin were randomly collected and were compared with parental populations. The PAP testing results showed that, among the 455 CRKP strains, 6.2% (28/455 isolates) were heteroresistant to colistin, which was similar to the frequency in the United States (8.4% [24/286 isolates]) (6). The frequency ranged from 1.0 × 10^−6^ to 5.9 × 10^−3^. Further susceptibility testing showed that the heteroresistant subpopulations exhibited greater colistin resistance than did their susceptible parental populations (Table 1).

**TABLE 1:** Characteristics of the 28 colistin-heteroresistant CRKP isolates studied

Isolate	Source	Frequency	MIC of resistant colonies (mg/L)	MIC of resistant colonies with CCCP (mg/L)	Fold decrease in MIC with CCCP	ST	Capsule type	Virulence gene(s)	Carbapenemase	ESBL and AmpC	*mcr-1*
HD1223	Blood	6.7 × 10^−5^	64	4	16	ST432	KL62	No	KPC-2	No	No
HD1511	Respiratory tract	3.7 × 10^−5^	32	0.5	64	Unknown	KL113	No	NDM-5	No	No
HD1513	Respiratory tract	3.4 × 10^−5^	32	<0.25	>128	ST11	KL64	No	KPC-2	CTX-M-65	No
HD1515	Respiratory tract	2.2 × 10^−5^	32	<0.25	>128	ST11	KL64	No	KPC-2	CTX-M-65	No
HD1517	Respiratory tract	5.0 × 10^−5^	32	<0.25	>128	ST11	KL64	No	KPC-2	CTX-M-65	No
HD1519	Respiratory tract	6.9 × 10^−5^	32	<0.25	>128	ST5216	KL144	No	IMP-4	No	No
HD1573	Blood	2.3 × 10^−6^	64	4	16	ST11	KL47	No	KPC-2	CTX-M-65	No
HD1698	Respiratory tract	5.4 × 10^−4^	128	4	32	Unknown	KL107	*iroB*	NDM-5	No	No
HD1706	Blood	2.0 × 10^−5^	64	<0.25	>256	ST11	KL64	No	KPC-2	CTX-M-65, SHV-12	No
HD1728	Blood	1.2 × 10^−5^	64	0.5	128	ST11	KL64	No	KPC-2	CTX-M-65, SHV-12	No
HD1752	Abdominal cavity	1.2 × 10^−6^	128	4	32	ST11	KL64	*iucA*, *rmpA2*, *iroB*	KPC-2	CTX-M-65	No
HD1867	Urinary tract	5.1 × 10^−6^	256	4	64	ST11	KL64	No	KPC-2	CTX-M-65	No
HD1911	Blood	1.1 × 10^−6^	128	4	32	ST11	KL64	No	KPC-2	CTX-M-65	No
HD1964	Respiratory tract	2.4 × 10^−5^	>256	4	>64	ST5216	KL144	No	IMP-4	No	No
HD1966	Respiratory tract	2.1 × 10^−5^	32	<0.25	>128	ST5216	KL144	No	IMP-4	No	No
HD1992	Urinary tract	7.8 × 10^−5^	64	4	16	ST5216	KL144	No	IMP-4	No	No
HD2000	Blood	2.5 × 10^−5^	128	4	32	ST5216	KL144	No	IMP-4	No	No
HD2008	Respiratory tract	2.2 × 10^−6^	64	2	32	ST1582	KL123	No	KPC-2	No	No
HD2286	Respiratory tract	2.6 × 10^−5^	32	4	8	ST11	KL64	No	KPC-2	SHV-12, CTX-M-65	No
HD2336	Respiratory tract	1.3 × 10^−6^	128	4	32	ST11	KL64	No	KPC-2	CTX-M-65	No
HD2575	Respiratory tract	5.2 × 10^−5^	64	2	32	ST5216	KL144	No	IMP-4	No	No
HD4333	Respiratory tract	2.0 × 10^−6^	128	2	64	ST11	KL64	No	KPC-2	CTX-M-65	No
HD4347	Blood	5.9 × 10^−3^	128	4	32	ST726	KL28	No	NDM-5	No	No
HD5523	Respiratory tract	1.6 × 10^−6^	64	4	16	ST11	KL64	*iucA*, *rmpA2*	KPC-2	CTX-M-65	No
HD5553	Urinary tract	1.0 × 10^−6^	64	4	16	ST11	KL64	*iucA*, *rmpA2*	KPC-2	CTX-M-65	No
HD5614	Respiratory tract	1.3 × 10^−6^	256	4	64	ST11	KL64	No	KPC-2	SHV-12	No
HD5628	Respiratory tract	2.1 × 10^−6^	64	2	32	ST11	KL64	No	KPC-2	SHV-12	No
HD6917	Respiratory tract	3.8 × 10^−6^	128	4	32	ST11	KL64	No	KPC-2	No	No

To examine the effects of efflux pumps on colistin heteroresistance, MICs were measured via the BMD method with the addition of 10 mg/L carbonyl cyanide *m*-chlorophenylhydrazone (CCCP) (an efflux pump inhibitor) to cation-adjusted Mueller-Hinton broth (CAMHB) (12). Colistin MICs with and without CCCP treatment and the fold decrease were calculated for each colistin-heteroresistant subpopulation. Results showed that all subpopulations had ≥8-fold decreases in MICs in the presence of CCCP, compared with those without CCCP (Table 1). Our results showed that the colistin heteroresistance in CRKP strains could be suppressed by CCCP, indicating that efflux pumps were involved in regulating colistin heteroresistance.

**FIG 1:**
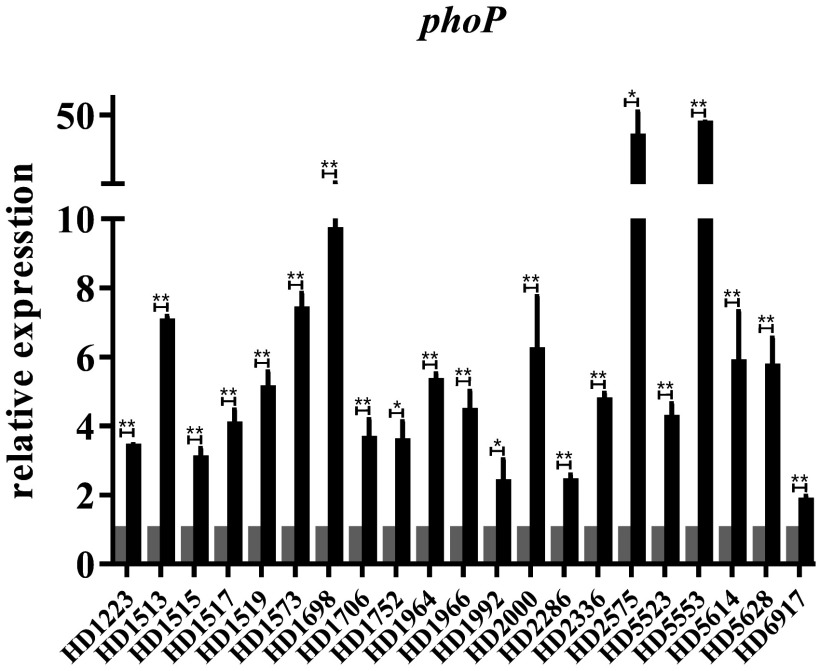
qRT-PCR analysis of *phoP* expression in heteroresistant and parental susceptible subpopulations. Relative expression was calculated by normalization to the expression of the 16S rRNA gene. *, *P* < 0.05; **, *P* < 0.01 (unpaired *t* test). The bars of gray colour were parental susceptible subpopulations, and black colour were heteroresistant strains.

To investigate more thoroughly the phenotypic differences between heteroresistant subpopulations and parental susceptible subpopulations, we quantified the expression of the two genes in the PhoPQ two-component system pathway, which reduced susceptibility to colistin through cationic sugar modifications to the lipid A component of lipopolysaccharide (LPS) (13). Total RNA samples from heteroresistant and parental susceptible isolates were extracted from a log-phase bacterial inoculum using TRIzol reagent (Sigma). Differences in gene expression were normalized to 16S rRNA gene expression and calculated by the 2^−ΔΔ^*^CT^* method. The primers for 16S rRNA gene expression (forward, GTGGGGAGCAAACAGGATTA; reverse, TAAGGTTCTTCGCGTTGCTT) were designed by Shanghai Sangon Biotech, while the primers for *phoP* expression were obtained from a previous study (14). All of the quantitative real-time PCRs (qRT-PCRs) were repeated at least three times. The results showed that *phoP* was upregulated in the 21 heteroresistant isolates, compared to its expression in parental susceptible isolates, indicating that the PhoPQ pathway played an important role in colistin heteroresistance (Fig. 1).

We wondered whether there was a difference in the colistin heteroresistance rates among the different hospitals. Among the six hospitals, the frequency of colistin heteroresistance ranged from 2.9% to 13.9%, with Guangzhou (13.9% [5/36 isolates]) having the highest rate and Ningxia (2.9% [2/69 isolates]) the lowest. The heteroresistance rates for the other four hospitals were 10.5% (Chengdu [11/105 isolates]), 6.3% (Beijing [2/32 isolates]), 3.7% (Suzhou [5/134 isolates]), and 3.8% (Kunming [3/79 isolates]). The rates of colistin heteroresistance were 3.3% (1/30 isolates) in 2016, 7.5% (20/268 isolates) in 2017, 6.9% (6/87 isolates) in 2018, and 1.4% (1/70 isolates) in 2019, and there was no statistical significance in the heteroresistance rates in different years (*P > *0.05) (Table 2).

**TABLE 2:** Area and year of isolation and colistin heteroresistance rates

Area and year of isolation	No. of isolates (%)			*P* ^*a*^
	Total (*n* = 455)	Heteroresistant (*n* = 28)	Nonheteroresistant (*n* = 427)	
Area				
Suzhou	134	5 (17.9)	129 (30.1)	0.165
Beijing	32	2 (7.1)	30 (7.0)	1.000
Chengdu	105	11 (39.3)	94 (22.0)	0.036
Guangzhou	36	5 (17.9)	31 (7.3)	0.099
Kunming	79	3 (10.7)	76 (17.8)	0.483
Ningxia	69	2 (7.1)	67 (15.8)	0.342
Year				
2016	30	1 (3.6)	29 (6.8)	0.786
2017	268	20 (71.4)	248 (58.1)	0.164
2018	87	6 (21.4)	81 (19.0)	0.749
2019	70	1 (3.6)	69 (16.1)	0.129

a *P* value for the colistin heteroresistance rate in each category, by odds ratio.

Next-generation sequencing was performed for the 28 heteroresistant isolates using a paired-end library, with an average insert size of 350 bp, on a NovaSeq 6000 system (Illumina, San Diego, CA, USA). The raw reads were assembled *de novo* utilizing SPAdes v3.11 (https://cab.spbu.ru/software/spades). Sequence types (STs) were determined using Kleborate (15), and the capsular types were determined using Kaptive (16). Antibiotic resistance genes in the genome assemblies were identified with ResFinder v4.1 (https://cge.food.dtu.dk/services/ResFinder). Virulence factors were defined using the Virulence Factor Database (VFDB) (http://www.mgc.ac.cn/VFs/main.htm).

**FIG 2:**
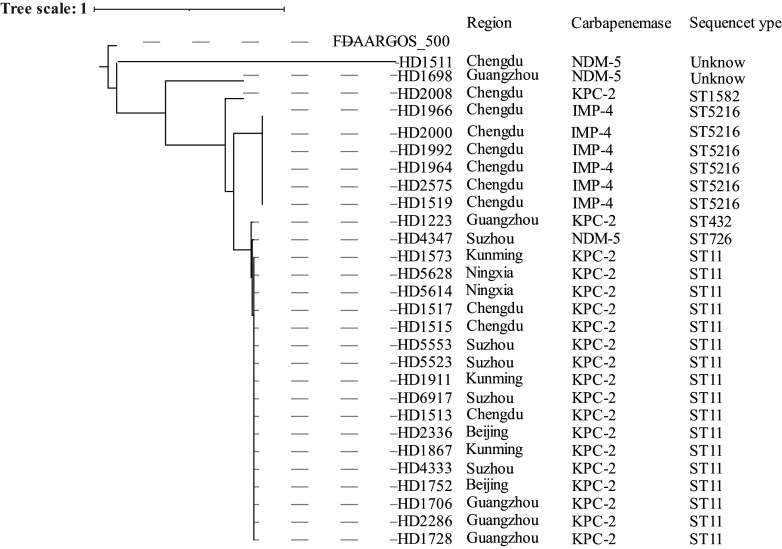
Maximum likelihood phylogenetic tree of colistin-heteroresistant strains. Bootstrap values for each cluster of associated taxa are shown next to the branches. The tree scale: 1 and a bootstrap iteration of 500.

We further investigated the STs of colistin-heteroresistant strains among CRKP strains (Fig. 2). ST11 (*n* = 17 [60.7%]) and ST5216 (*n* = 6 [21.4%]) were the predominant STs, and other STs included ST432 (*n* = 1 [3.6%]), ST726 (*n* = 1 [3.6%]), ST1582 (*n* = 1 [3.6%]), and two novel STs. ST11 strains were found in six tertiary care hospitals, while the ST5216 strains were isolated from the same hospital (Fig. 2). Core single-nucleotide polymorphisms (SNPs) were identified with MUMmer v3.25 (17). A maximum likelihood phylogenetic tree was constructed using MEGA X v10.1.8 (18), based on the core SNPs with a bootstrap iteration of 500, and plotted using iTOL (https://itol.embl.de). We performed clonal relatedness analysis based on SNPs for the ST5216 and ST11 strains. Among the partial ST11 strains, there were 0 to 3 SNP differences between different cities. Among the 6 ST5216 strains, there were 2 SNP differences, suggesting a common origin.

In our results, carbapenemase genes were detected in all 28 heteroresistant isolates (Table 1). Among them, three types of carbapenemases genes were detected, i.e., *bla*_KPC-2_ (*n* = 19 [67.9%]), *bla*_IMP-4_ (*n* = 6 [21.4%]), and *bla*_NDM-5_ (*n* = 3 [10.7%]). The 6 ST5216 strains carried *bla*_IMP-4_, and 17 ST11 strains carried *bla*_KPC-2_. Sixteen isolates also carried extended-spectrum β-lactamases (ESBLs), mostly CTX-M-65. Worryingly, 4 isolates (HD1867, HD1752, HD5523, and HD5553) were positive by the string test, which has been strongly associated with hypervirulence. In addition, 4 strains (HD1698, HD1752, HD5523, and HD5553) were found to harbor at least one of the *iucA*, *rmpA2*, and *iroB* genes, which have been regarded as the typical markers of hypervirulent K. pneumoniae (HvKP) (19).

Colistin is generally chosen as one of the last-resort antibiotics to treat carbapenem-resistant *Enterobacteriaceae* infections. Importantly, colistin heteroresistance can lead to antibiotic treatment failure in the clinic. This is the first large multicenter surveillance study in China for colistin heteroresistance among CRKP strains. Our study enriches the epidemiological study of colistin heteroresistance in China, emphasizing that colistin heteroresistance is an underestimated phenomenon among CRKP strains.

### Data availability.

The draft whole-genome sequences for the 28 heteroresistant isolates have been deposited in GenBank under BioProject accession number PRJNA809827.
